# How Computations Can Assist the Rational Design of Drugs for Photodynamic Therapy: Photosensitizing Activity Assessment of a Ru(II)-BODIPY Assembly

**DOI:** 10.3390/molecules27175635

**Published:** 2022-09-01

**Authors:** Fortuna Ponte, Davide Maria Scopelliti, Nico Sanna, Emilia Sicilia, Gloria Mazzone

**Affiliations:** 1 Dipartimento di Chimica e Tecnologie Chimiche, Università della Calabria, 87036 Rende, CS, Italy; 2 Department for Innovation in Biology Agro-Food and Forest Systems (DIBAF), University of Tuscia, Largo dell’Università snc, 01100 Viterbo, VT, Italy

**Keywords:** BODIPY, Ru(II) complex, conjugate, PDT, DFT, photosensitizer

## Abstract

Ruthenium-based complexes represent a new frontier in light-mediated therapeutic strategies against cancer. Here, a density functional-theory-based computational investigation, of the photophysical properties of a conjugate BODIPY-Ru(II) complex, is presented. Such a complex was reported to be a good photosensitizer for photodynamic therapy (PDT), successfully integrating the qualities of a NIR-absorbing distyryl-BODIPY dye and a PDT-active [Ru(bpy)_3_]^2+^ moiety. Therefore, the behaviour of the conjugate BODIPY-Ru(II) complex was compared with those of the metal-free BODIPY chromophore and the Ru(II) complex. Absorptions spectra, excitation energies of both singlet and triplet states as well as spin–orbit-matrix elements (SOCs) were used to rationalise the experimentally observed different activities of the three potential chromophores. The outcomes evidence a limited participation of the Ru moiety in the ISC processes that justifies the small SOCs obtained for the conjugate. A plausible explanation was provided combining the computational results with the experimental evidences.

## 1. Introduction

Despite the great efforts made in cancer research so far, standard cancer therapies still face major challenges, mainly stemming from their low specificity towards cancer cells over the healthy ones. Several strategies were proposed to overcome these drawbacks, and *in situ* activation of the cytotoxic drugs is one of these. As light can be conveyed with very high spatiotemporal control, it is well suited for this purpose. Photodynamic therapy (PDT) is a light-based technique that, due to its excellent features, such as spatiotemporal selectivity, non-invasiveness, minimal drug resistance and reduced side effects, is well-established and clinically applied for cancer treatment. The therapy relies on the use of therapeutic agents, called photosensitizers (PSs), able to produce toxic reactive oxygen species (ROS), singlet oxygen ^1^O_2_ and free radicals that cause cell death [1,2]. The PS, when localised in cancer cells, is excited from the ground state to an exited singlet state, by irradiation with a laser light of wavelength comprised in the range 600–850 nm [3,4], also known as the therapeutic window. This step is followed by either decay to the ground state or by intersystem crossing (ISC) radiationless transition to a low-lying triplet state. The transition between the two electronic states of different multiplicity is usually forbidden by the selection rules, but the ISC process is driven by spin–orbit coupling (SOC) between the two electronic states. The triplet excited state can decay to the ground state via a light emission process (phosphorescence) or undergo two kinds of photochemical reactions, called type I and type II [5,6]. Type I reactions involve the formation of free radicals and radical ions mediated by organic substrates that, reacting with molecular oxygen, lead to the formation of reactive oxygen species (i.e., O_2_^−•^, HO^•^, H_2_O_2_), able to damage the DNA structure [7,8,9]. The photochemical reactions of type II, instead, produce the highly reactive singlet oxygen, ^1^O_2_, due to the energy transfer from the PS triplet state to the molecular oxygen ground state (^3^O_2_)_._ The cytotoxic agent ^1^O_2_ oxidizes a wide range of biomolecules in cells, ultimately inducing the death of cancer cells via a combination of necrosis and/or apoptosis [10].

So far, different classes of PSs were proposed and used in the PDT field. The most studied and successfully applied PSs are porphyrin-based drugs. Recently, on the basis of experimental and theoretical studies [11,12,13], new compounds, belonging to the family widely known as boron dipyrromethenes (BODIPYs), were proposed as promising candidates for PDT due to their ability to generate singlet oxygen, high solubility in common organic solvents and very favourable chemical–physical properties [14]. Although BODIPYs, due to their remarkable fluorescence quantum yields, usually exhibit negligible triplet-state formation, their possible derivatization allows the fine modulation of the photophysical behaviour. In particular, to enhance the ISC process and promote the spin−orbit coupling, BODIPY-based derivatives are generally designed introducing heavy atoms such as halogens (i.e., iodine or bromine) in different numbers and positions or transition metal atoms to generate metal–chromophore conjugates [11,15]. The combination of BODIPYs with metal complexes garnered considerable attention over the last few years [16,17]. The synthesis of these systems, which show a good photostability, is versatile and, due to the metal presence, other electronic transitions, i.e., MLCT, LMCT and d-d transitions, become accessible beside the diagnostic π-π* and n-π* transitions. The complexes containing metal atoms such as Pt(II), Pt(IV), Os(II), Re(I), Ir(III) and Ru(II), owing to the high probability of triplet-state formation and tunable photophysical, photochemical and redox properties are promising candidates for PDT, photochemotherapy (PCT) and photoactivated chemotherapy (PACT) [18,19,20,21,22]. The research, currently conducted in the PDT area, is mainly focused on the design of platinum and ruthenium BODIPY systems. In spite of the numerous examples of Pt(II) and Pt(IV) complexes combined with BODIPYs reported in the literature [16,17,23,24,25,26], the most studied metal-based compounds in this field are undoubtedly Ru(II)-containing systems. These complexes are generally considered less toxic with respect to the platinum-based drugs, show high chemical stability and photostability, high ^1^O_2_ production and appear to follow a different mechanism of action. In particular, Ru(II)-polypyridyl complexes are the most studied in PDT and PCT [27,28,29,30] and, worthy of note, the McFarland’s photodrug TLD-1433 was the first Ru-based photodrug to enter in a human clinical trial phase II as a novel PDT photosensitizer (ClinicalTrials.gov Identifier NCT03945162) for the treatment of bladder cancer [2].

**Scheme 1:**
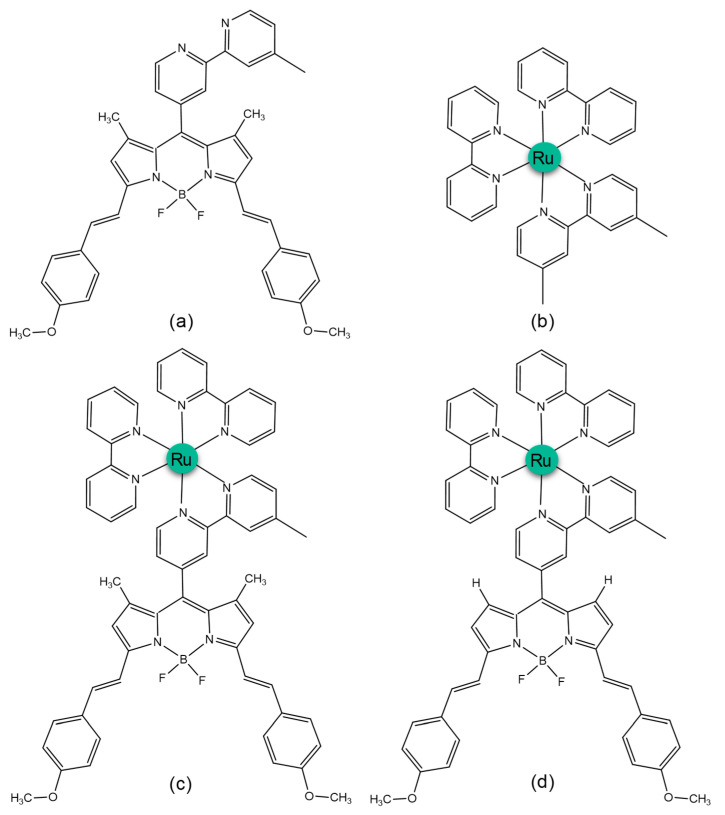
Schematic representation of the investigated molecules (**a**) BDP (**b**) Ru (**c**) Ru-BDP and (**d**) Ru-HBDP.

Despite their remarkable properties, most of the ruthenium polypyridyl complexes are activated in PDT by blue or UV light, consequently limiting the possibility to treat deep or large tumours. In particular, the lowest energy absorption band in these complexes is mainly attributed to an MLCT transition that, generally, falls in the region 400−500 nm [31]. In order to overcome these limits, and thereby increasing the range of treatable cancers, many efforts were focused on developing systems that exhibit a red-shifted maximum absorption wavelength (λ_max_). One way to shift λ_max_ is the coordination of ligands with extended π-systems just such as BODIPYs [32]. This strategy allows for prolonging the lifetime of the Ru(II) triplet excited state due to a greater ISC and to obtain a higher singlet oxygen yield. Recently, the [Ru(bpy)_3_]^2+^ complex, active in PDT, was used to functionalize a distyryl-BODIPY with the aim to obtain NIR-driven photosensitization. Together with the synthesized Ru(II)-BODIPY conjugate system, here reported as Ru-BDP (see Scheme 1), both its components, the metal-free BODIPY, named BDP, and the Ru complex (indicated as Ru), were tested in vitro and in vivo [33]. The authors have demonstrated that the Ru-BDP system, exploiting simultaneously the favourable properties of its components, is a potent candidate for a new generation of PDT agents acting in the proper NIR region. Specifically, the authors intended to exploit the direct linkage between the two subunits in order to allow full electronic communication and maximise the heavy atom effect (HAE). Therefore, the BDP absorption in the NIR region should be combined with the Ru efficient production of singlet oxygen in the Ru(II)-BODIPY conjugate. Additionally, the authors observed that the very low cellular uptake of the separated subunits corresponds to an enhanced uptake of the conjugated complex.

Density functional theory (DFT) and its time-dependent extension (TD-DFT) allow to study the electronic structure in the ground and excited states and to reliably calculate the excitation energies and spin–orbit coupling (SOC) constants, through conjugating accuracy and computational cost [34,35]. Such tools were used here to explore this proposed conjugation aiming at clarifying how the presence of the Ru(II) moiety can affect the photochemical behaviour of the BODIPY dye. Furthermore, the photophysical properties of a Ru-BODIPY system, in which methyl groups in 1,7 positions of the BODIPY core were substituted with H atoms, were calculated in order to verify whether the decrease in the steric hindrance of such substituents could allow a proper reciprocal arrangement that favours the conjugation between the Ru(II) moiety and the BODIPY dye.

## 2. Results and Discussion

For a molecule to be used in PDT, a series of chemical and photophysical features have to be satisfied. These include: strong absorption in the therapeutic window to allow tissue penetration; low-lying triplet energy larger than 0.98 eV, that is the amount of energy required to promote the transition of molecular oxygen from the triplet state to the singlet excited one; and high SOC values that ensure an efficient ISC process. In this perspective, BODIPYs, thanks to their interesting structural, optical and photophysical features, such as an intense absorption band in the visible region, represent one the most studied classes of compounds for PDT application [36]. The BODPY scaffold permits a fine derivatization so that chemical modifications can easily tune the spectroscopic properties. In particular, extensive studies were devoted to clarifying the influence of *meso*-substitution [37,38]. In the present study, the BODIPY under consideration presents two structural modifications: the inclusion in *meso* position of a 4-methyl-2,2’-bipyridine in order to extend the π system, and two styryl substituents, with electron-donating properties, bound to the BDP core (Scheme 1) [33]. In the search for more active PSs, the BDP core was appended to a Ru(II) complex (Ru), characterised by two bipyridine ligands and a 4,4′-dimethyl-2,2′-bipyridine.

In order to highlight the difference between the precursors, BDP and Ru, with the conjugate complex, Ru-BDP, the photophysical properties of the three compounds were firstly investigated and described in detail in the following section. Moreover, on the basis of the obtained results that do not confirm the role that should be played by the conjugation between BDP and Ru in enhancing ISC kinetics, a more accurate study was performed in order to search for the key properties of the synthesized Ru-BDP complex to be able to better photosensitize ^1^O_2_ with respect to its separated precursors.

### 2.1. Photophysical Properties of BDP, Ru and Ru-BDP Conjugate

**Figure 1:**
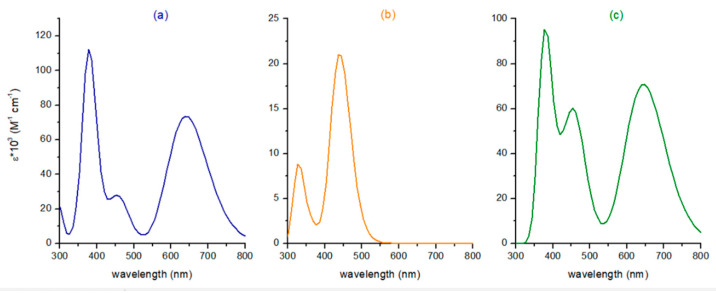
Calculated UV–vis absorption spectra of (**a**) BDP, (**b**) Ru, and (**c**) Ru-BDP in water at B3LYP/6-31+G* (SDD for Ru) level of theory.

To accurately describe the photophysical properties of the systems under investigation, a preliminary benchmark study was carried out on the BDP absorption spectrum and compared with an experimentally available one obtained in acetonitrile solvent [33]. The appropriate protocol was selected on the basis of the reproduction of the maximum absorption wavelength (λ_max_), one of the key parameters for PDT application. The performance of several exchange and correlation functionals was evaluated and the results are collected in Table S1. B3LYP functional was, thus, selected for all the calculations carried out on the systems under investigation. In particular, the calculated λ_max_ for BDP is in excellent agreement with the experimental value (644 and 641 nm, respectively). The calculated UV–vis spectra for the two precursors, BDP and Ru, and for the Ru-BDP conjugate, computed in the solvent water to mimic the physiological environment, are reported in Figure 1.

For all the systems under investigation, only singlet excited states with an oscillator strength value (*f*) higher than 0.01, considered to be essential in determining the different absorption bands, are reported in Table 1. The percentage composition of molecular orbitals for each of the reported transitions is also included in the same table.

The calculated absorption spectrum of BDP is comprised essentially of four bands, three high-energy bands in the range 320–420 nm and a low energy band around 550–750 nm (Figure 1). According to the general behaviour of BODIPYs, the band in the near UV region is the most intense, but it is not suitable for PDT application since it falls outside the therapeutic window. The dye possesses a significant red maximum absorption at 641 nm, corresponding to the first transition (Tr = 1 in Table 1) with a π-π* character and attributed to an exclusively HOMO (H)→LUMO (L) excitation (100%). Furthermore, the hole/particle orbitals, based on the natural transition orbitals’ (NTOs) plots, are mainly localised on the styryl groups (Figure S1). The other broad bands appearing in the UV region display an intense intra-ligand charge-transfer, ILCT, character. The most intense band is centred to the transition calculated at 379 nm, with the highest oscillator strength, and is mainly originated by H→L+2, H-1→L excitations.

Additionally, the photophysical properties of the metal chromophore, Ru, were investigated here. Ru absorbs light in the UV–visible region, and the most intense transitions are included in Table 1. The spectrum is characterised by an intense absorption band not suitable for the treatment of deep tumours since it falls in the spectral range 400–500 nm. Such a band is characterised by transitions with different features, which are detailed in Table 1. It is centred at 436 nm, and low-energy NTOs (Figure S2) for 1–5 transitions show that it is a mixed band with MLCT and ILCT characters. The excitation to the S_1_ state (Tr = 1), corresponding to the H→L transition, with a calculated λ_max_ of 502 nm, has an almost zero oscillator strength and therefore little physical significance. TD-DFT assigns the most intense transition (Tr = 5), with the highest oscillator strength and originated by H−2→L+1 and H−1→L+2 MOs, to the bright singlet state at 436 nm. The NTOs inspection evidences almost pure MLCT character for such a state. A second, weaker absorption band, of appreciable intensity, between 300–380 nm and centred at 326 nm, can be observed. The band is attributed to spin-allowed MLCT mixed to ILCT excitations, as evidenced by the NTOs collected in Figure S2.

Finally, in order to determine the influence of the Ru complex on the photophysics of the BDP dye, the electronic spectrum of the Ru-BDP conjugate was compared with those of the two components. As it is possible to infer from the absorption spectra shown in Figure 1, the presence of the ruthenium metal complex linked to the BDP core only slightly influences the shape of the spectral features, especially in the low-energy region, the most important for PDT application. Indeed, the position of the maximum absorption band is shifted from 641 nm in BDP to 645 nm in Ru-BDP. As in the BDP system, even for Ru-BDP, the λ_max_ of the band observed in the red/NIR region is characterised by a transition, here indicated as ILCT, involving the distyryl-BODIPY moiety. In addition, the spectrum of the Ru-BDP conjugate shows two absorption bands in the range 320–500 nm of appreciable intensity. The band centred at the transition Tr = 3 (H-4→L, H-2→L+1) calculated at around 463 nm, is a mix of different transitions with ILCT, MLCT and LMCT characters (Figure S3). The other band detected below 400 nm is peaked at 378 nm and has a significant LLCT character.

To be able to exert the PS activity, the chromophore must be able to promote an ISC from a bright state or low-lying singlet state and must have a triplet state with energy higher than the amount of energy required to excite molecular oxygen from its ground triplet state to the singlet one, computed to be 0.91 eV at the B3LYP/6-31G* level of theory [39]. The energies and the character of all the triplet states with energy lower than the bright one are reported in Table 2. While for BDP S1 is the bright state (1.93 eV) and only one triplet state lies below (1.01 eV), for the Ru complex there are nine triplet states with energy lower than the bright state. Despite not being explicitly reported in Table 1, all the singlet states lying below the bright one, resulted to be the S8, were taken into consideration for ISC process feasibility. Interestingly, the S1 state turns out to be the bright one in the Ru-BDP conjugate, confirming the main spectral features are dictated by the BDP unit even in the presence of the appended metal complex. The NTOs computed for each triplet state of the precursors as well as of the conjugate are collected in Figure S4. Therefore, while for BDP and Ru-BDP S1→T1 is the only accessible channel for ISC to occur, in Ru, all the singlet states lying below the S8 bright one could be, in principle, involved in triggering ISC processes. Table 3 reports the computed SOC values and the energy gaps (in brackets) between the coupled states that could play a role in the radiationless ISC pathways.

S1 and T1 states are separated by only 0.92 eV in the case of BDP, and the SOC calculated for the radiationless transition to occur is 0.02 cm^−1^. The two states are essentially originated by the same electronic transition (H→L) and, thus, the ISC has to occur among states with very similar characters. Differently, the Ru complex, as with most metal-containing systems, possess a series of excited singlet states that are originated by charge-transfer from metal to ligand (MLCT) and vice versa (LMCT). This means that the transition from singlet state to triplet manifold can involve a change of orbital type, thus resulting in a large rate of ISC, as postulated by the El-Sayed rule [40].

As properly underlined in the computational methods section, ISC kinetics directly depends on the amplitude of the spin–orbit-matrix elements. Thus, the SOC values computed for all the plausible ISC channels of Ru (Table 3) starting from the bright state, (S8) and admitting that even internal conversion (IC) processes between states with the same multiplicity can occur, several couplings can return a large rate of ISC, see e.g. SOC for S2→T3, S5→T2 and so on. Even taking into consideration only the coupling of the states separated by a little energy difference, ΔE, as in the case of S3→T6 or S5→T8, the ISC results in being highly favourable. Therefore, based on these evidences, the Ru complex is very active in photosensitizing ^1^O_2_.

**Figure 2:**
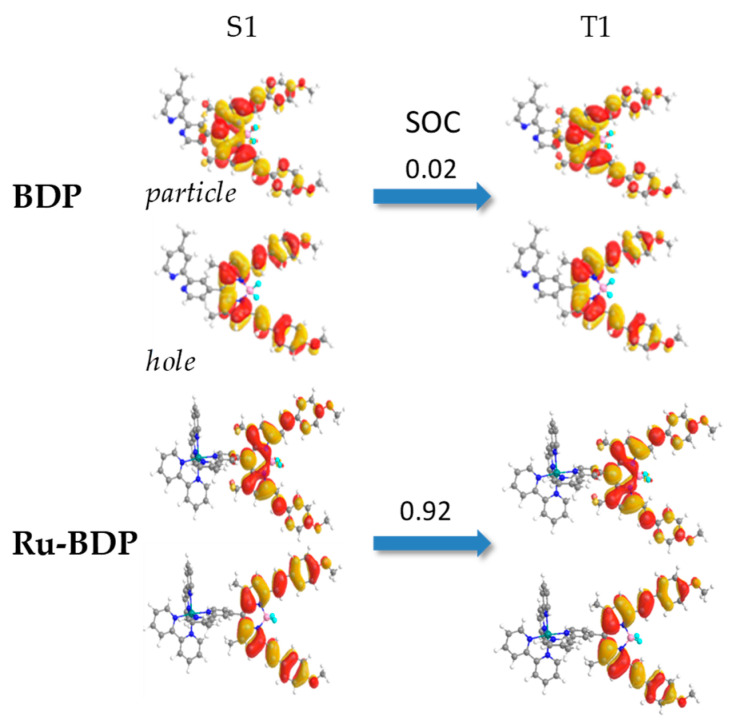
Natural transition orbitals (hole and particle) computed for S1 and T1 of BDP and Ru-BDP. SOC values in cm^−1^ are also provided.

On the other hand, the conjugate complex presents only one triplet state (T1) with energy lower than the bright singlet one (S1), and the radiationless transition among these states separated by 0.92 eV is accomplished with an SOC value of 0.92 cm^−1^. Though the SOC value is one order of magnitude greater than that of BDP, it is not comparable with those obtained for the Ru complex. In addition, a very similar behaviour of Ru-BDP and BDP was found in regards to the properties for evaluating ISC efficiency. As underlined above, the maximum absorption wavelength and then the first singlet excited state is insensitive to the metal presence. Indeed, the NTOs associated to the same state in the two cases are originated by the same orbitals, as evidenced in Figure 2. At the same time, even the triplet state is centred on the BDP in the two cases, so a very weak HAE could be admitted.

### 2.2. Structural Features of the Conjugate

From the data obtained for the precursors, BDP and Ru, it appears evident that the presence of the metal in the conjugate does not significantly enhance ISC kinetics. In fact, the computed SOC for the only plausible radiationless transition (S1→T1) remains quite similar to that computed for the BDP chromophore. Looking at the NTO plots obtained for the two involved excited states in Ru-BDP (Figure 2), there is no participation of the metal in both states, leaving the radiationless transition entirely centred on the BDP chromophore.

**Figure 3:**
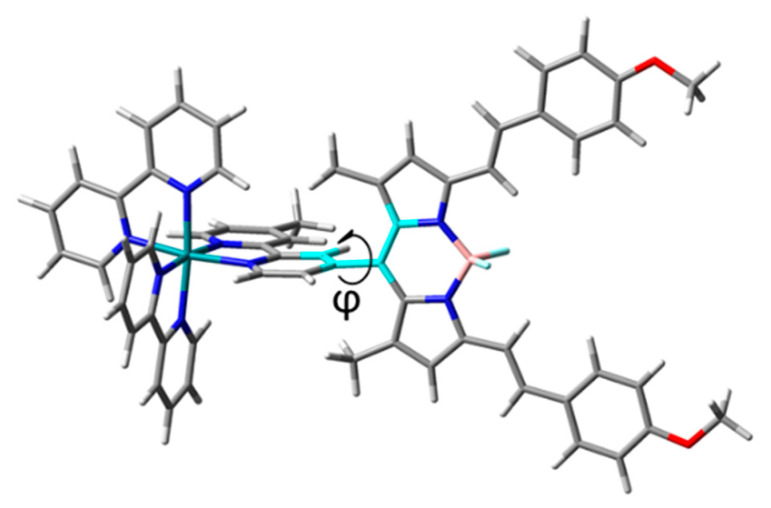
Optimised structure of the Ru-BDP conjugate.

**Figure 4:**
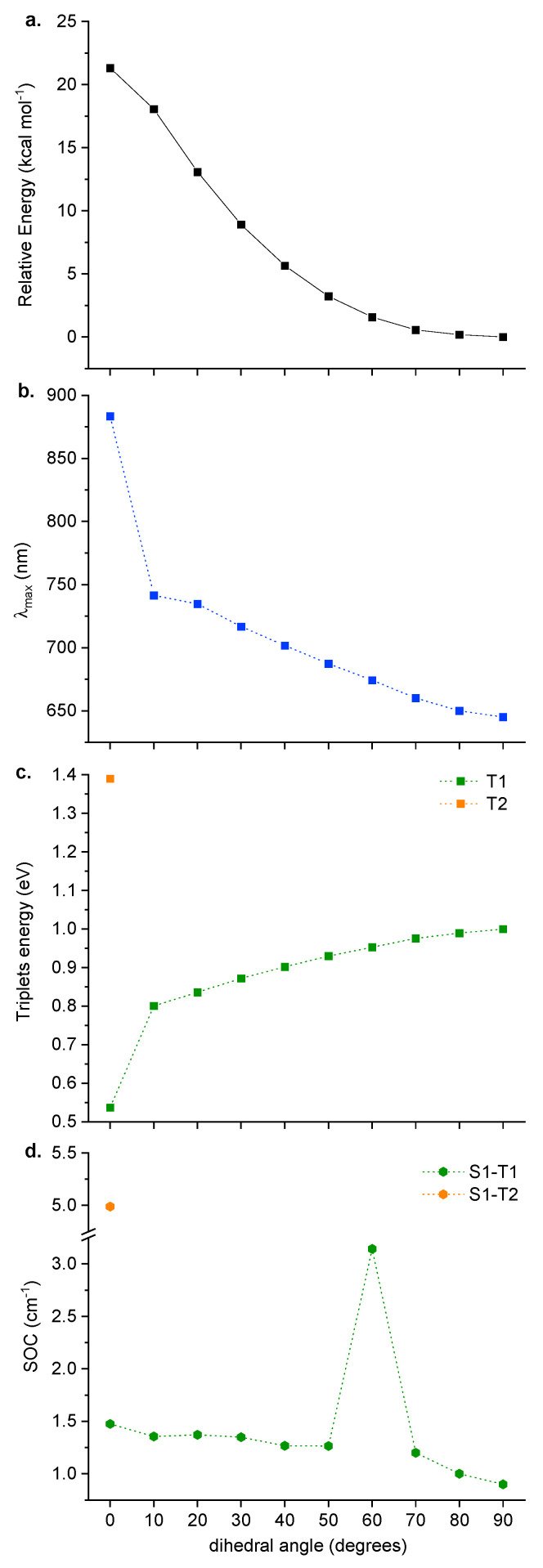
(**a**) relative energy of each scan point with respect to the most stable semi-relaxed structure, (**b**) maximum absorption wavelength, (**c**) triplet states energy and (**d**) SOCs computed for the accessible ISC channels.

As properly underlined by the authors in their experimental exploration [33], to exert a synergic photodynamic action, the Ru complex must be linked to the BDP unit in a way to maximise the conjugation. That means the BDP core and the bipy ligand of the Ru complex should be approximately coplanar for a full electronic communication to occur. Analysing the optimised structure of the conjugate, as reported in Figure 3, it is evident that the bipy and the BDP core are approximately orthogonal to each other (torsion angle φ equal to 87.5 degrees). This feature is, very likely, responsible for the breaking of the conjugation that hampers the communication between the chromophore and the heavy atom. All the attempts to find an alternative structure in which the two key portions of the photosensitizer could be almost coplanar failed. Thereby, we performed a semi-relaxed scan calculation by exploring all the PDT-related properties as a function of the variation of the torsion angle, highlighted in Figure 3, in the attempt to obtain a constrained structure characterised by a proper coupling between the two components of the conjugate and to compute its key properties. The outcomes of this exploration are collected in Figure 4 and Table S2, where the relative energy with respect to the most stable semi-relaxed structure is calculated for each point of the scan calculation. Maximum absorption wavelength, triplet states energy and SOCs computed for the accessible ISC channels are also provided.

Reading the data starting from the angle closest to that of the absolute minimum, which is 90 degrees, the result worthy of note is surely the decrease in energy of the low-lying triplet state as the torsion angle decreases. Therefore, the possibility for the PS to transfer its triplet-state energy to molecular oxygen, which requires 0.91 eV to be excited, is prevented. So, below 40 degrees, it happens that the conjugate becomes inactive in PDT. In contrast, with the decrease in the φ dihedral angle, the maximum absorption wavelength increases up to more than 850 nm because of the extension of the conjugation that at 0 degrees involves the bipy portion and even the Ru metal centre. Interestingly, the conformation with φ = 0 is the only one for which two triplet states lie below the bright singlet one. Therefore, differently from all the other arrangements, there are two possible ISC channels, S1→T1 and S1→T2. However, the T1 energy is too small, and it is unreasonable it could be involved in the energy transfer required for Type II reactions to occur. On the other hand, the computed S1→T2 SOC value becomes 5 cm^−1^. Indeed, the NTO analysis evidences a direct bipy and Ru participation in both the hole and particle of the T2 state and only to the particle of the S1 state (Figure S5), which means there is a variation in the orbital type upon transition that should ensure fast ISC kinetics.

Looking at all the SOC values in Figure 4d, it can be noted that the decrease in the φ angle corresponds to a gradually increasing participation of the metal to the NTOs explaining the slight increased value for the S1→T1 coupling (see Figure S5). However, the obtained values remain in the order of a few cm^−1^ because both S1 and T1 states are generated from the same electronic transition and the associated NTOs are quite similar. Only the value obtained for φ = 60 becomes two times those computed for all the other arrangements, at least for the S1→T1 radiationless transition. It is characterised by a slightly major participation of the metal atom to the particle isodensity plot (Figure S5). So, it seems to be the right value for a good coupling between the metal centre and the BDP chromophore. In addition, it is only 1.6 kcal mol^−1^ less stable than the minimum, therefore easily accessible. In regard to the other parameter that mostly weighs in ISC kinetics, [41] the singlet–triplet energy splitting among the coupled states ΔE_S-T_, it does not vary so much, changing the dihedral angle. From 0 to 60 degrees, it remains in the range 0.85–0.89 eV, while increases up to 0.93 eV for the structure at 90 degrees (Table S2). Therefore, even looking at the energy splitting it is difficult to discriminate among the various structural arrangements. The only noteworthy difference along the dihedral angle scan remains the SOC value computed for the S1→T1 coupling in the 60 degrees arrangement.

For that reason, we imagined that groups less hindered than methyl in 1,7 positions, even if the BDP and the *meso* bipy group are not coplanar, should allow an electronic communication between the chromophore and the heavy atom to ultimately enhance ISC kinetics. Then, we have replaced the methyl groups with H atoms, naming Ru-HBDP the modified complex, in order to minimise the steric hindrance that compromises the conjugation. The corresponding TD-DFT calculations are collected in Table S3 and Figure S6. The spectrum has a fairly similar shape to that theoretically recorded for Ru-BDP. Ru-HBDP shows the most intense absorption band at 378 nm with an LMCT character. A pronounced shoulder covers the range 430–600 nm with ILCT, involving the BDP portion, MLCT and LMCT characters. The most important band for PDT application is centred at 686 nm and is red-shifted by 45 nm with respect to BDP reference (641 nm), suggesting a different behaviour with respect to that found for the Ru-BDP conjugate, for which the maximum absorption wavelength remains essentially that of BDP. Looking at the data in Table S2, it can be seen that the red-shifted band is dominated by the H→L transition. According to the NTOs’ character reported in Figure S6, the band indicates a direct coupling of the metal centre with the π-system of the distyryl-BODIPY moiety, showing a significant LMCT character. However, similar to Ru-BDP, even in this case the bright state remains the S1, and only one triplet state, T1, lies below it, and they are separated by 0.89 eV. Moreover, the S1 and T1 states present the same character, as they are both originated by the H→L electronic transition, thus the ISC cannot be accompanied by a change of molecular orbital type. Indeed, the computed SOC value remains in the order of a few cm^−1^ (1.43). Notably, despite the limited steric hindrance of the hydrogen atoms, the obtained structure is characterised by a torsion angle, accounting for the reciprocal position of bipy and BDP moieties, of 53.8 degrees. The PDT-related properties are, indeed, similar to those computed for the forced torsion angle of 50 degrees of Ru-BDP.

The results reported here evidence a weak HAE in the proposed conjugate, which suggests that the enhanced singlet oxygen quantum yield found for Ru-BDP (0.77) with respect to BDP (0.00) and Ru (0.66) should be ascribed to other factors such as the improved drug uptake due to a proper combination of charge and lipophilicity [33]. Indeed, good water solubility and promoted cellular uptake are characteristics underscored only for the conjugate, and these features could be responsible for the enhanced photosensitizing activity in vitro and in vivo of the proposed conjugate. Ru-BDP is, indeed, characterised by an optimal logP_o/w_ of −0.6, in contrast to BDP and Ru that present logP_o/w_ values of 3.1 and −1.3, respectively. 

It can be concluded, on the basis of all these evidences, that the better performance experimentally observed for the conjugate cannot be ascribed to the enhancement of ISC kinetics due to the presence of the Ru centre, but rather to a proper combination of charge and lipophilicity.

## 3. Computational Details

All the calculations presented in this paper were performed with Gaussian 16 suite of programs [42] employing DFT and time-dependent DFT methods.

Ground singlet state optimizations for BDP, Ru, Ru-BDP and Ru-HBDP were carried out by using the B3LYP functional [43,44] with Grimme’s D3 dispersion correction [45] and adopting the SMD implicit solvation model [46] to simulate an aqueous environment (ε = 80). The SDD effective core potential [47] and the corresponding valence basis set were selected for the metal atom, Ru, while 6-31 + G* basis set was used for all the other atoms.

Time-dependent DFT calculations, employing the same protocol, were carried out on the ground-state structure of all the systems under investigation to determine vertical excitation energies and to simulate the optical absorption spectra in water. For all the systems, twenty singlet and triplet excitations were calculated. The computational results are summarised in Table 1, Table 2 and Table S2, where only electronic transitions with an oscillator strength value (f) higher than 0.01 are reported.

This protocol was selected testing the performance of different DFT functionals through the comparison of the calculated maximum absorption wavelength with the corresponding experimental one detected for the BDP compound in an acetonitrile solvent [33]. B3LYP-D3 [43,44,45], B3PW91 [44,48,49], CAM-B3LYP-D3 [50], B97D [51], ϖB97XD [52], TPSS [53], PBE0 [54], PBE [55], M06 [56], M11 [57], MN12L [58], MN15 [59] and MN15L [60] were used for this purpose. Essentially, B3LYP-D3, B3PW91 and MN12L exhibited the best performance in the reproduction of the absorption data. Therefore, in order to maintain the same protocol used for the optimizations, B3LYP-D3 was selected for the exploration of the photophysical properties of all the investigated compounds.

To prove the occurrence of ISC processes, the spin–orbit-matrix elements for the coupling of the states potentially involved were calculated at the ground-state-optimised geometry of the investigated systems with ORCA code [61,62] and SOC values obtained, according to Equation (1):

(1) $$SOC_{nm}=\sqrt{\underset{i}{\sum }{\left|\langle \psi_{S_{n}}\left|{\hat{H}}_{SO}\right|\psi_{T_{i,m}}\rangle \right|}^{2}};i=x,y,z$$

where ${\hat{H}}_{SO}$ is the spin–orbit Hamiltonian with effective nuclear charge. Relativistic corrections were obtained by the zeroth order regular approximation (ZORA). ZORA-DEF2-SVP and SARC-ZORA-SVP were used for the main and metal atoms, respectively. The RIJCOSX approximations were introduced to speed up the calculations’ time, as suggested in the ORCA manual. Very tight SCF convergence and a very large grid (Lebedev 770 points) were set for such calculations.

To check whether use of the ground-state structure could affect ISC kinetics, for the Ru-BDP conjugate, SOC values were calculated considering also the first excited state structure (S1). The outcomes of these calculations are summarised in Figure S7. Even in the S1 excited state, the conformational arrangement of the conjugate again does not allow any electronic communication between the Ru complex and the BDP chromophore. Indeed, the computed SOC remains in the order of 1 cm^−1^. For this reason, for exploring the ISC process, the ground-state structure was considered for all the other SOC calculations.

## 4. Conclusions

Density functional theory and its time-dependent extension were employed here to explore the photophysical properties of a Ru-BODIPY conjugate, recently proposed as an effective photosensitizer for ^1^O_2_ generation in the framework of PDT. For the sake of comparison, the same properties were computed for its components, BDP dye and Ru complex. For this purpose, the electronic spectra as well as the triplet-state energies and spin–orbit-matrix elements for the accessible ISC channels were computed for each of them. The comparison between BDP and the conjugate Ru-BDP returns only a marginal participation of the Ru moiety in the key region of spectrum (600–850 nm), and analysing the excited state’s energy, only one ISC channel is possible in both cases, S1→T1. The computed SOC values in the two cases, 0.02 and 0.92 cm^−1^ for BDP and Ru-BDP, respectively, confirm, to some extent, the conclusion already evidenced by the poor shift of the maximum absorption wavelength that remains around 640 nm. On the other hand, the ISC-related quantities computed for the precursor Ru complex strongly confirm the potential role of Ru polypyridyl complexes in promoting the radiationless processes.

The structural arrangement of the conjugate shows that bipy and BDP are approximately orthogonal to each other. It is this arrangement that might be, presumably, responsible for the breaking of the conjugation among them hampering the electronic communication between the chromophore and the heavy atom. The exploration of the key photophysical properties, as a function of the variation of the torsion angle linking them, showed that a worthy conjugation should be found if BDP and bipy are at around 60 degrees, one to the other. Thus, the design of a new conjugate, Ru-HBDP system, in which methyl groups in 1,7 positions of the BDP core were replaced with H atoms, is proposed with the aim to limit the steric hindrance and then maximise the communication between the BDP dye and the heavy atom centre Ru. However, PDT-related properties of such a complex supported, again, only a marginal participation of the metal centre to the states, which are both generated by the same electronic transition, involved in the ISC process.

The results reported here prove that only a weak heavy-atom effect in the proposed conjugate exists. As the main photophysical properties of the Ru-BODIPY compound remain unchanged with respect to those of the BDP chromophore, it can be suggested that the favourable uptake of the drug, rather than the enhancement of ISC kinetics by Ru centre, is responsible for the better performance experimentally observed. Therefore, a proper functionalization of the distyryl-BODIPY for both enhancing its ISC, like the addition of heavy atoms directly linked to the chromophore core, and contemporarily reducing its logP_o/w_, for favouring its permeation through the phospholipidic membrane, could lead to an efficient photosensitizer without the inclusion of a metal complex.

## Figures and Tables

**Table 1:** Excitation energies (ΔE, eV), absorption wavelength (λ, nm), oscillator strength (*f*), MO contribution (%) for selected transitions (Tr) of BDP, Ru and Ru-BDP compounds.

Compound	Tr ^a^	Band	ΔE	λ	*f*	MO Contribution ^b^	Theoretical Assignment
BDP	1	I	1.93	641	1.04	H→L 100%	π-π*
	2	II	2.71	457	0.38	H-1→L 87%	π-π*
	3	III	3.02	410	0.08	H→L+1 89%	π-π*
	4		3.27	379	1.36	H→L+2 72%	
	5		3.28	378	0.17	H-2→L 49%, H-3→L 18%	
	6		3.53	351	0.04	H→L+4 59%, H→L+3 34%	
	7		3.56	348	0.05	H-5→L 99%	
	8		3.64	341	0.02	H→L+3 41%, H→L+4 36%	
	9	IV	4.16	298	0.03	H→L+6 85%	π-π*
	10		4.20	295	0.24	H-9→L 77%	
	11		4.21	294	0.06	H→L+7 71%	
Ru	1	I	2.47	502	0.00	H→L 92%	MLCT/ILCT
	2		2.67	464	0.01	H-1→L+1 52%, H-2→L 28%	
	3		2.74	451	0.06	H-2→L 62%, H-2→L+2 22%	
	4		2.83	438	0.08	H-2→L+2 67%, H-1→L+1 25%	
	5		2.85	436	0.14	H-2→L+1 46%, H-1→L+2 43%	MLCT
	6	II	3.31	374	0.01	H→L+3 98%	MLCT/ILCT
	7		3.49	355	0.01	H-1→L+3 98%	
	8		3.52	352	0.01	H-2→L+3 99%	
	9		3.66	338	0.01	H→L+5 97%	
	10		3.80	326	0.06	H-1→L+6 79%	
Ru-BDP	1	I	1.92	645	1.00	H→L 100%	ILCT
	2		2.33	532	0.01	H→L+1 84%	LMCT/ILCT
	3	II	2.68	463	0.49	H-4→L 47%, H-2→L+1 44%	ILCT
	4		2.69	462	0.07	H-3→L+1 45%, H-2→L+1 21%	LMCT/ILCT
	5		2.70	458	0.04	H-2→L+2 42%, H-2→L+1 18%	MLCT
	6		2.71	457	0.04	H-3→L+1 40%, H-3→L+3 25%	MLCT
	7		2.83	438	0.12	H-3→L+3 40%, H-2→L+2 26%	MLCT/ILCT
	8		2.85	436	0.13	H-2→L+3 36%, H-3→L+2 35%	MLCT
	9	III	3.04	408	0.19	H→L+4 85%	LLCT/ILCT
	10		3.28	378	1.27	H→L+5 76%	LLCT/ILCT

Tr = transition number. ^a^. only vertical transitions with oscillator strength greater than 0.01 are reported, with the exception of the most red-shifted transition. ^b^. only percentage greater than 18% are reported.

**Table 2:** Excitation energies (ΔE, eV), absorption wavelength (λ, nm), MO contribution (%) to the triplets excited states for BDP, Ru-m, Ru-BDP.

Compound	State	ΔE	MO Contribution	Theoretical Assignment
BDP	T1	1.01	H→L 99%	π-π*
Ru	T1	2.33	H→L+1 88%	MLCT/ILCT
	T2	2.34	H→L 63%, H→L+2 26%	MLCT/ILCT
	T3	2.40	H-2→L+1 62%, H-1→L+2 25%	MLCT/ILCT
	T4	2.41	H→L+2 61%, H→L 27%	MLCT
	T5	2.48	H-1→L+1 40%, H-2→L 39%	MLCT/ILCT
	T6	2.53	H-1→L+2 68%, H-2→L+1 25%	MLCT
	T7	2.58	H-1→L 85%	MLCT/ILCT
	T8	2.60	H-1→L+1 46%, H-2→L 44%	MLCT/ILCT
	T9	2.70	H-2→L+2 74%	MLCT/LLCT
Ru-BDP	T1	1.00	H→L 99%	ILCT

**Table 3:** Computed SOCs (cm^−1^) for S_n_-T_m_ radiationless transitions and singlet-triplet splitting energies, ΔE (eV), between the involved states in brackets.

		Tm								
	**Sn**	1	2	3	4	5	6	7	8	9
BDP	1	0.02 (0.92)								
Ru	1	64.31 (0.14)	17.43 (0.13)	174.79 (0.07)	3.92 (0.06)					
	2	35.10 (0.17)	59.63 (0.16)	326.53 (0.10)	8.99 (0.09)	277.95 (0.02)				
	3	24.47 (0.25)	26.99 (0.24)	143.18 (0.18)	61.56 (0.16)	74.62 (0.09)	316.15 (0.04)			
	4	323.15 (0.34)	220.03 (0.33)	269.23 (0.27)	7.92 (0.26)	4.39 (0.19)	62.45 (0.14)	72.67 (0.09)	41.38 (0.07)	
	5	144.13 (0.34)	339.81 (0.33)	9.60 (0.28)	138.15 (0.26)	267.93 (0.19)	28.83 (0.14)	17.97 (0.09)	131.45 (0.07)	
	6	51.57 (0.42)	65.83 (0.41)	59.51 (0.35)	378.67 (0.34)	16.05 (0.27)	143.54 (0.22)	239.22 (0.17)	11.13 (0.14)	27.29 (0.05)
	7	222.18 (0.51)	313.33 (0.49)	6.87 (0.44)	97.27 (0.42)	37.20 (0.35)	270.91 (0.30)	146.0 (0.25)	38.06 (0.23)	27.92 (0.14)
	8	256.01 (0.52)	35.11 (0.51)	3.48 (0.45)	294.79 (0.43)	155.84 (0.37)	31.32 (0.31)	28.09 (0.27)	179.42 (0.24)	161.21 (0.15)
Ru-BDP	1	0.92 (0.92)								

## Data Availability

Not applicable.

## Supplementary Materials

The following supporting information can be downloaded at: https://www.mdpi.com/article/10.3390/molecules27175635/s1, Table S1. TD-DFT benchmark for BDP chromophore on the structure optimised at B3LYP/6-31 + G** level in CH_3_CN implicit solvent; Figure S1. Natural Transition Orbitals (NTOs), hole and particle, for transitions labelled 1–11 for BDP system; Figure S2. NTOs for transitions labelled 1–10 for Ru complex; Figure S3. NTOs for transitions labelled 1–10 for Ru-BDP complex; Figure S4. NTOs for triplet states of BDP, Ru and Ru-BDP systems lying below the bright singlet one; Table S2. PDT-related data for each scan point of the torsion angle variation in the Ru-BDP complex; Figure S5. NTOs for the bright singlet state (S1) and low-lying triplet states obtained for each scan point of the torsion angle variation in the Ru-BDP complex; Table S3. Excitation energies (ΔE, eV), absorption wavelength (λ, nm), MO contribution (%) to selected transition for Ru-HBDP and for the T1 state; Figure S6. Computed absorption spectrum for Ru-HBDP, NTOs for transitions labelled 1–13 and for the triplet state (T1) lying below the bright one. Figure S7. S1 excited state structure and related SOC(S1-T1).
