# Supplementary material for: How Computations Can Assist the Rational Design of Drugs for Photodynamic Therapy: Photosensitizing Activity Assessment of a Ru(II)-BODIPY Assembly
Source: Molecules. 2022 Sep 1;27(17):5635. doi: 10.3390/molecules27175635 (PMC9457801; doi:10.3390/molecules27175635)
Supplement: Supplementary file 1 [file molecules-27-05635-s001.zip › molecules-1832619-supplementary.pdf]

Supplementary Materials

How computations can assist the rational design of drugs for photodynamic therapy: photosensitizing activity assessment of a Ru(II)-BODIPY assembly

Fortuna Ponte[1]*, Davide Maria Scopelliti[1], Nico Sanna[2], Emilia Sicilia[1] and Gloria Mazzone[1]*

[1]Dipartimento di Chimica e Tecnologie Chimiche, Università della Calabria, 87036 Rende, CS, Italy;

[2]Department for Innovation in Biology Agro-Food and Forest Systems (DIBAF), University of Tuscia, Largo dell'Università snc, 01100 Viterbo, Italy;

Table of Contents

Table S1. TD-DFT benchmark for BDP chromophore on the structure optimized at B3LYP/6-31+G** level in CH_3CN implicit solvent. **S2**

Figure S1. Natural Transition Orbitals (NTOs), hole and particle, for transitions labelled 1-11 for **BDP** system. **S3**

Figure S2. NTOs for transitions labelled 1-10 for Ru complex. **S4**

Figure S3. NTOs for transitions labelled 1-10 for Ru-BDP complex. **S5**

Figure S4. NTOs for triplet states of BDP, Ru and Ru-BDP systems lying below the bright singlet one. **S6**

Table S2. PDT-related data for each scan point of the torsion angle variation in the Ru-BDP complex. **S7**

Figure S5. NTOs for the bright singlet state (S1) and low-lying triplet states obtained for each scan point of the torsion angle variation in the Ru-BDP complex. **S8**

Table S3. Excitation energies (ΔE, eV), absorption wavelength (λ, nm), MO contribution (%) to selected transition for Ru-HBDP and for the T1 state. **S9**

Figure S6. Computed absorption spectrum for Ru-HBDP, NTOs for transitions labelled 1-13 and for the triplet state (T1) lying below the bright one. **S10**

Figure S7. S1 excited state structure and related SOC(S1-T1) **S12**

References **S13**

Table S1

	λ	eV	f	MO contribution[a]	λ_{exp} [q]
B3LYP-D3	644	1.93	1.0358	H→L, 99%	641
B3PW91	642	1.93	1.0386	H→L, 100%	
camB3LYP-D3	594	2.09	1.1050	H→L, 96%	
B97D	705	1.76	0.9437	H→L,99%	
ωB97XD	587	2.11	1.1106	H→L,94%	
TPSS	693	1.79	0.9653	H→L,99%	
PBE0	630	1.97	1.0632	H→L,100%	
PBE	710	1.75	0.9289	H→L,99%	
M06	639	1.94	1.0452	H→L,100%	
M11	578	2.15	1.0900	H→L,92%	
MN12L	640	1.94	1.1056	H→L,99%	
MN15	627	2.01	1.0619	H→L,98%	
MN15L	637	1.95		H→L,100%	

[a] data in CH_3CN from ref [1].

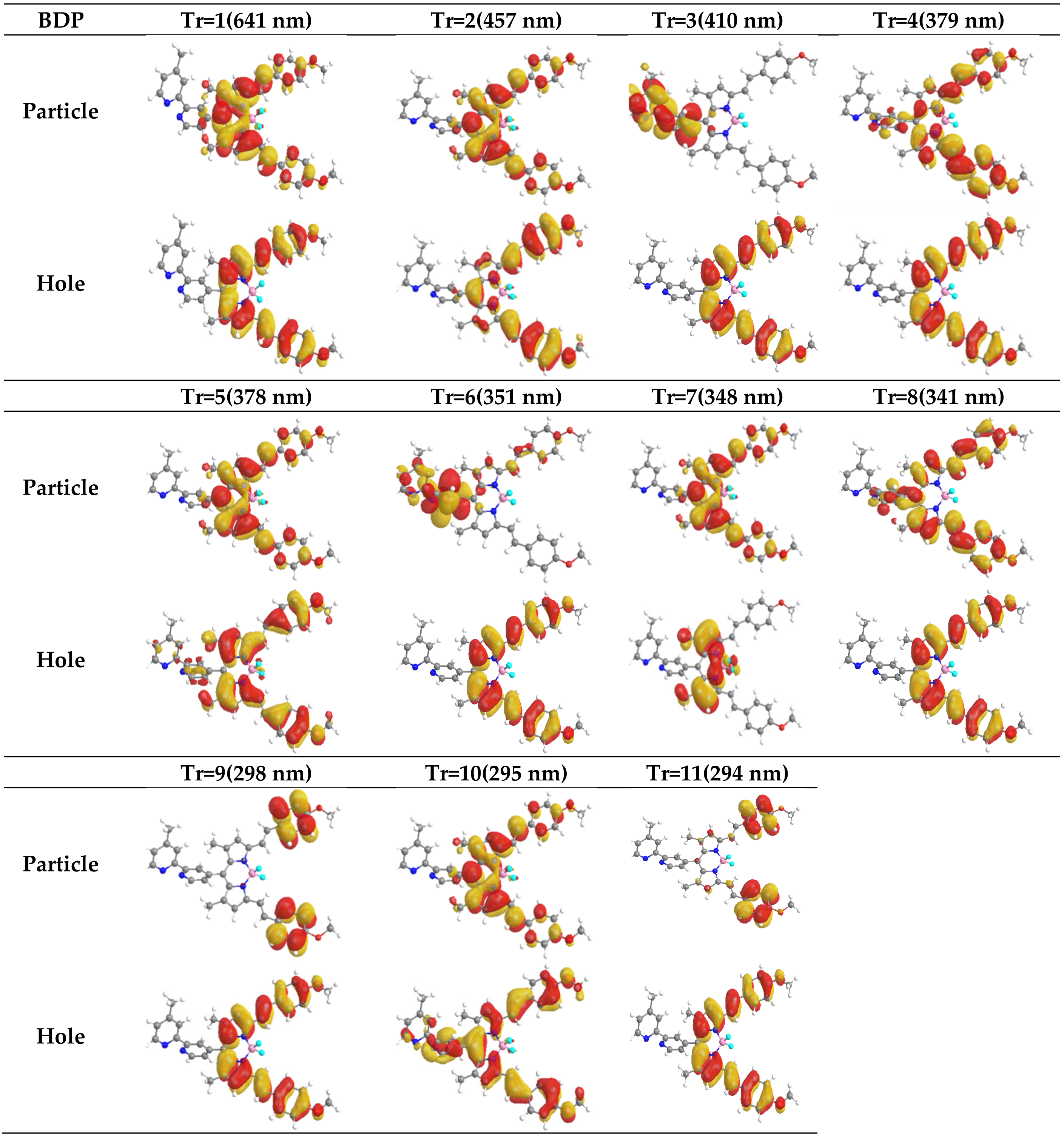
BDP
Tr=1(641 nm)
Tr=2(457 nm)
Tr=3(410 nm)
Tr=4(379 nm)
Particle
Hole
Tr=5(378 nm)
Tr=6(351 nm)
Tr=7(348 nm)
Tr=8(341 nm)
Particle
Hole
Tr=9(298 nm)
Tr=10(295 nm)
Tr=11(294 nm)
Particle
Hole

Figure S1

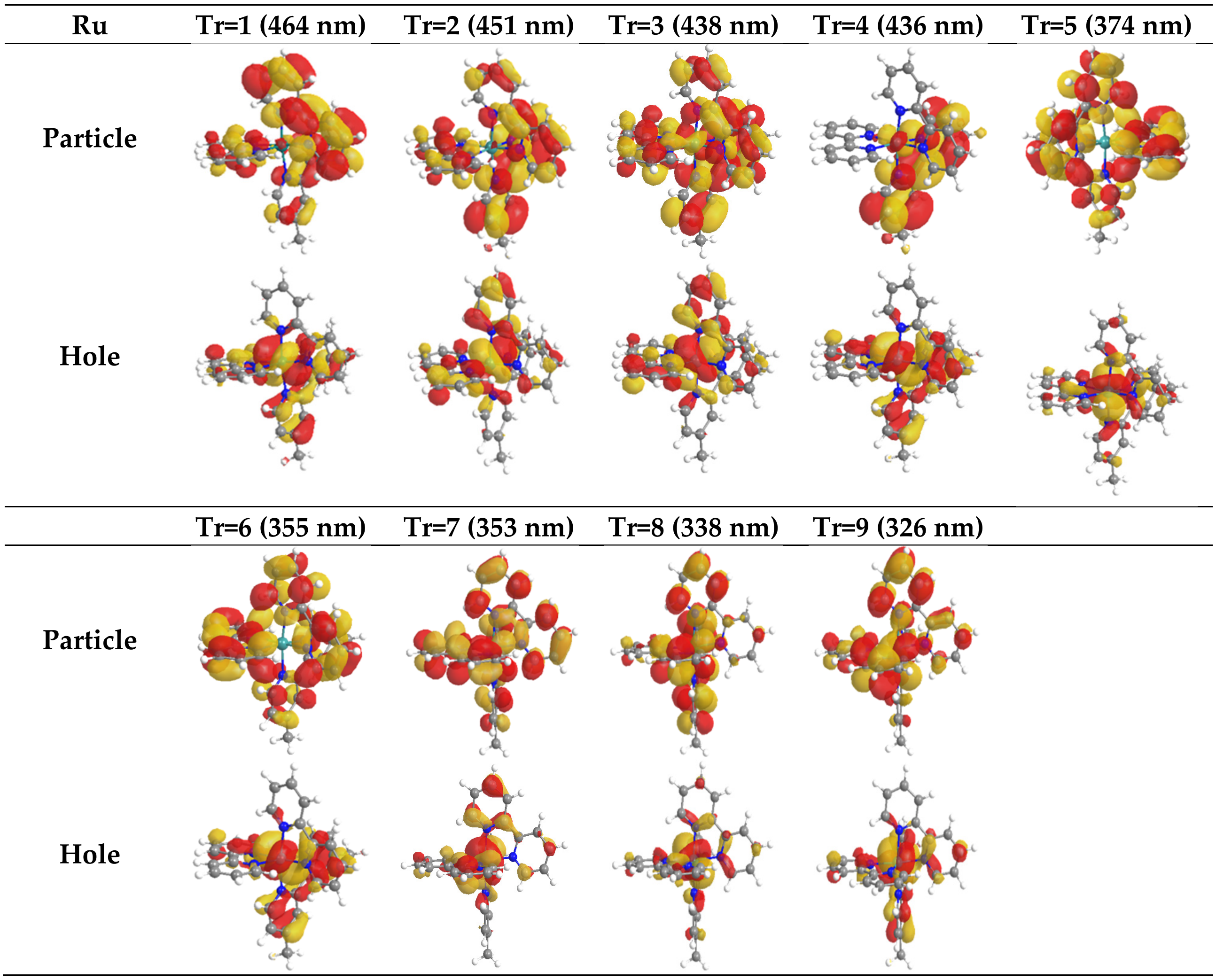
Ru
Tr=1 (464 nm)
Tr=2 (451 nm)
Tr=3 (438 nm)
Tr=4 (436 nm)
Tr=5 (374 nm)
Particle
Hole
Tr=6 (355 nm)
Tr=7 (353 nm)
Tr=8 (338 nm)
Tr=9 (326 nm)
Particle
Hole

Figure S2

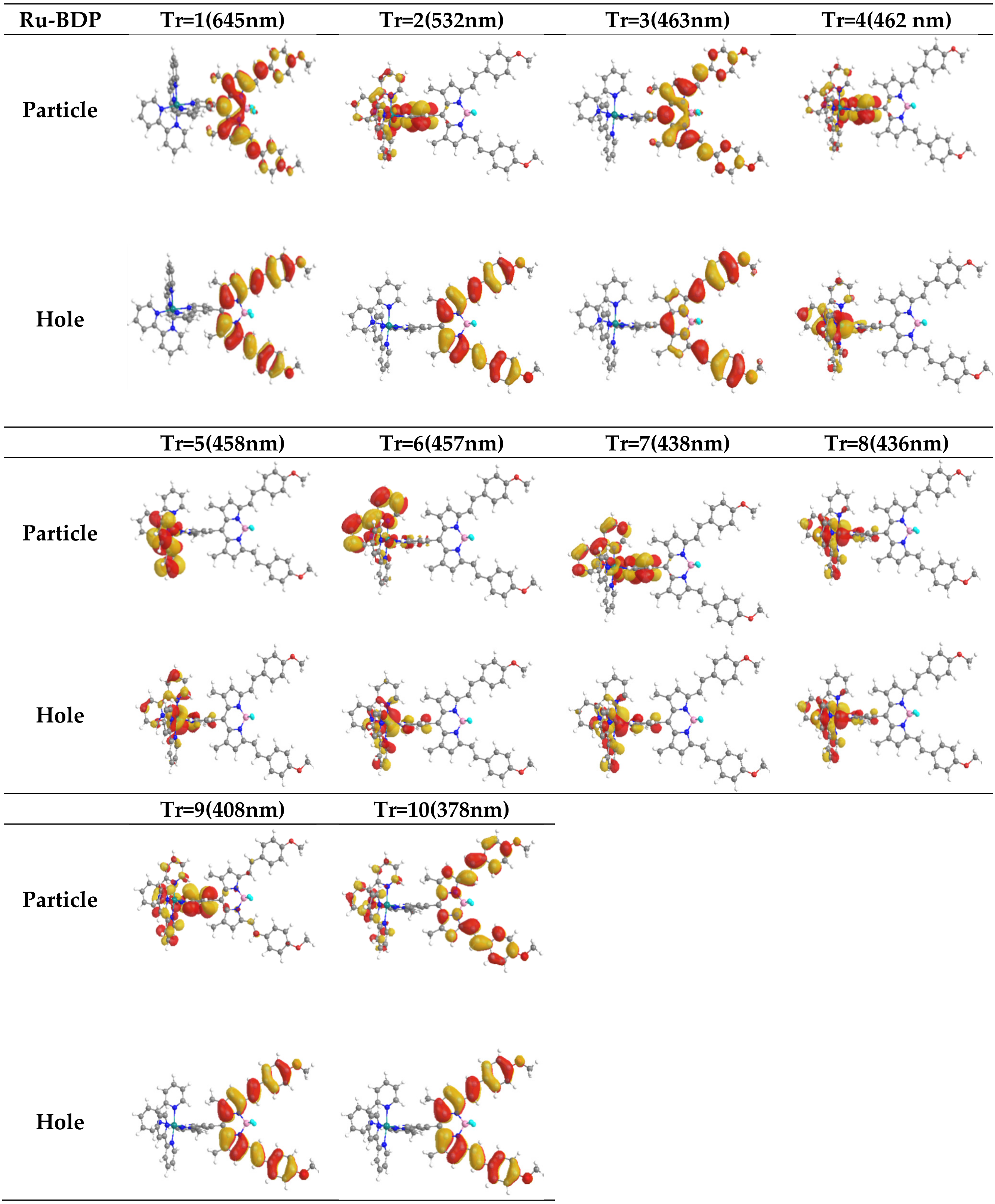
Ru-BDP
Tr=1(645nm)
Tr=2(532nm)
Tr=3(463nm)
Tr=4(462 nm)
Particle
Hole
Tr=5(458nm)
Tr=6(457nm)
Tr=7(438nm)
Tr=8(436nm)
Particle
Hole
Tr=9(408nm)
Tr=10(378nm)
Particle
Hole

Figure S3

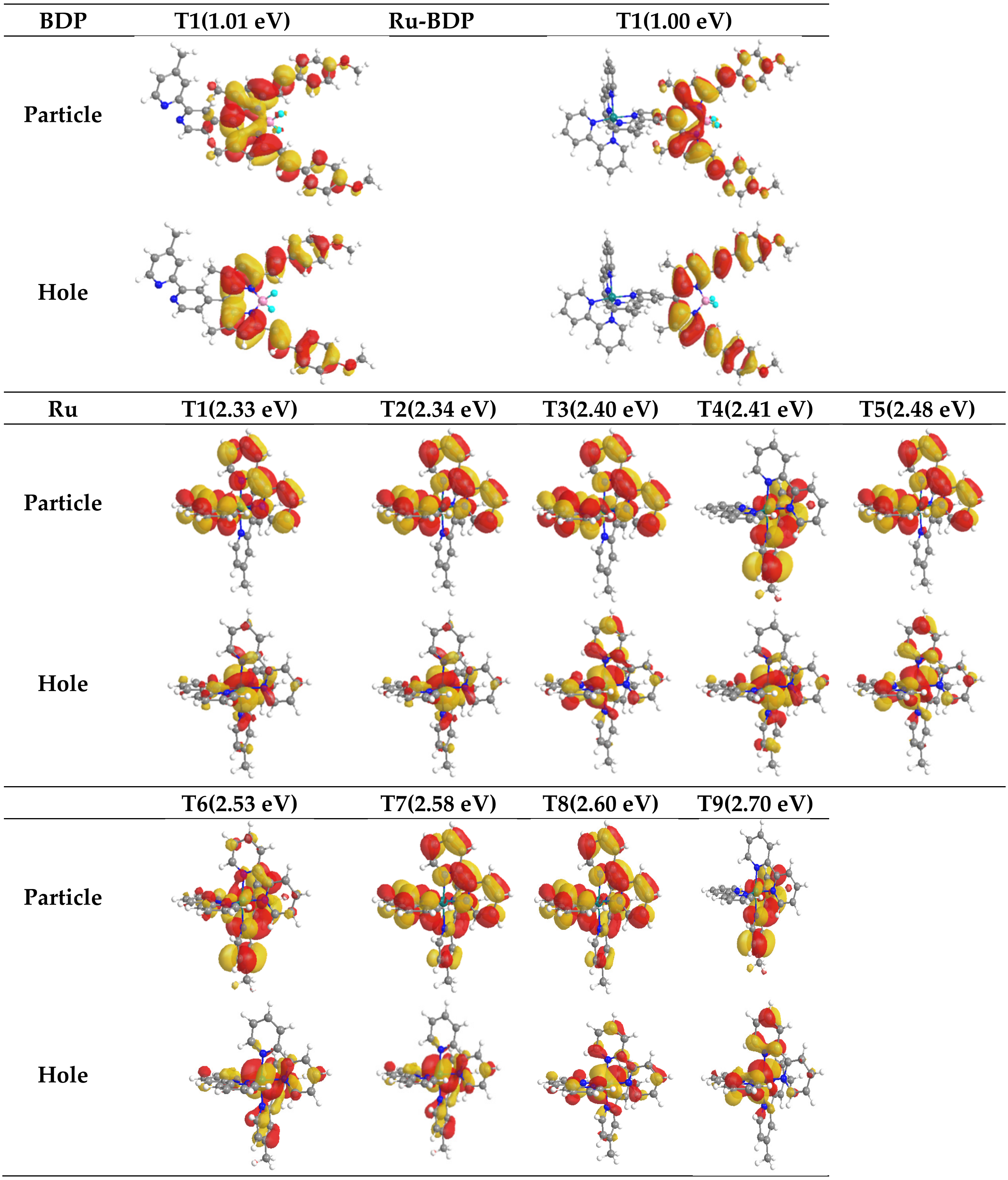
BDP
T1(1.01 eV)
Ru-BDP
T1(1.00 eV)
Particle
Hole
Ru
T1(2.33 eV)
T2(2.34 eV)
T3(2.40 eV)
T4(2.41 eV)
T5(2.48 eV)
Particle
Hole
T6(2.53 eV)
T7(2.58 eV)
T8(2.60 eV)
T9(2.70 eV)
Particle
Hole

Figure S4

Table S2

Angle	E_{rel} (kcal mol^{-1})	λ^{max} (nm)	T1,T2 (eV)	ΔE_{S1-Tm} (eV)	SOC (cm^{-1})
0	21.3	883	0.54, 1.39	0.86, 0.0	1.5, 5.0
10	18.1	741	0.80	0.85	1.4
20	13.1	735	0.84	0.85	1.4
30	8.9	717	0.87	0.86	1.3
40	5.6	702	0.90	0.86	1.3
50	3.2	687	0.93	0.87	1.3
60	1.6	674	0.95	0.89	3.1
70	0.6	660	0.98	0.90	1.2
80	0.2	650	0.99	0.92	1.0
90	0.0	645	1.00	0.93	0.9

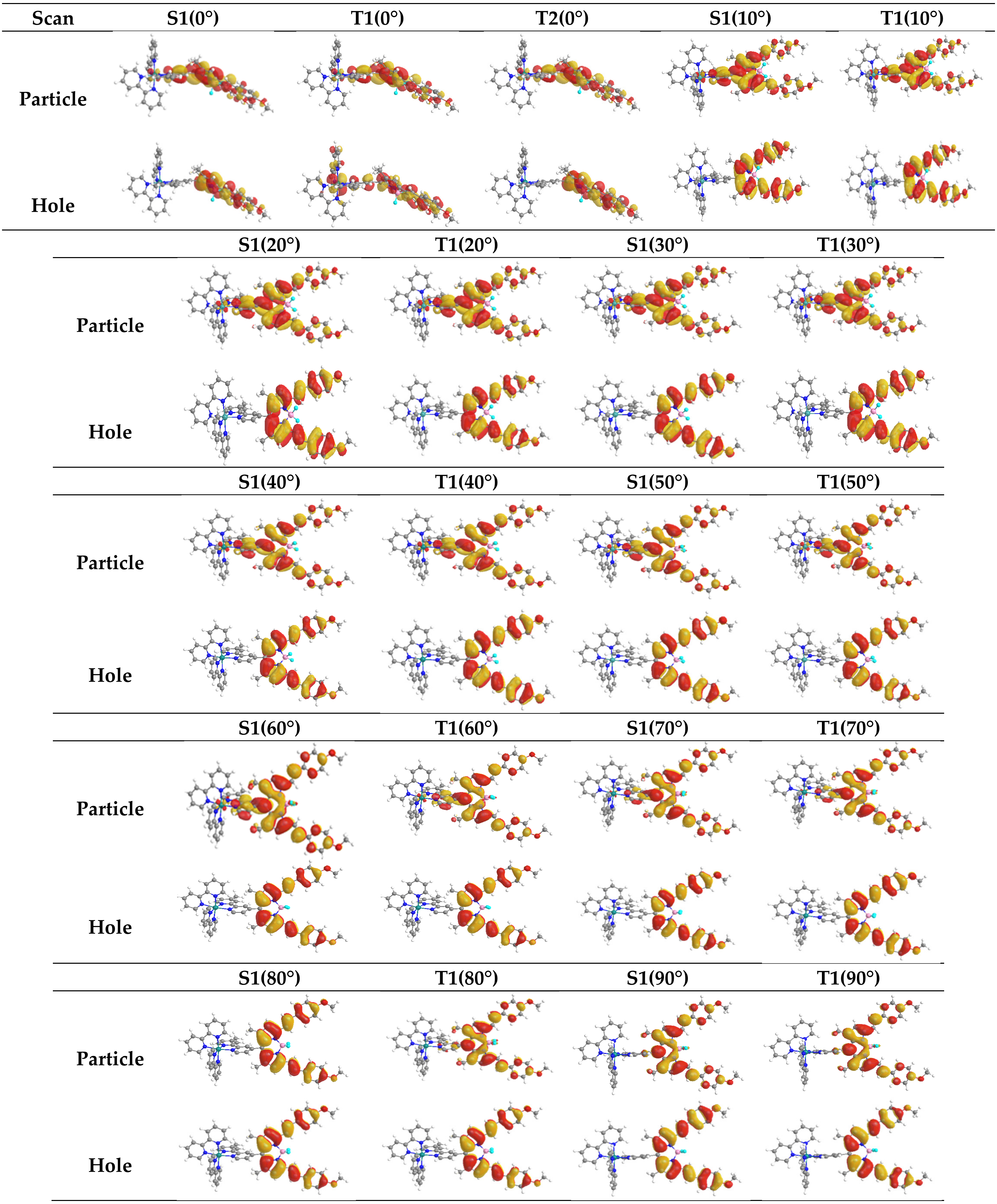
Scan
S1(0°)
T1(0°)
T2(0°)
S1(10°)
T1(10°)
Particle
Hole
S1(20°)
T1(20°)
S1(30°)
T1(30°)
Particle
Hole
S1(40°)
T1(40°)
S1(50°)
T1(50°)
Particle
Hole
S1(60°)
T1(60°)
S1(70°)
T1(70°)
Particle
Hole
S1(80°)
T1(80°)
S1(90°)
T1(90°)
Particle
Hole

Figure S5

Table S3

Ru-HBDP

Tr[a]	Band	State	ΔE	λ	f	MO contribution	Theoretical Assignment
1	I	S1	1.81	686	0.89	H→L 100%	LMCT
2	II		2.10	591	0.05	H-1→L 92%	MLCT/ LMCT
3			2.27	545	0.45	H-2→L 96%	
4			2.45	505	0.06	H→L+1 78%	
5			2.58	481	0.36	H-4→L 89%	
6			2.60	477	0.02	H→L+3 52%, H→L+2 27%, H→L+1 20%,	
7			2.72	455	0.01	H-2→L+1 45%, H-2→L+2 16%, H-2→L+3 14%	
8			2.80	443	0.07	H-3→L+1 59%, H-2→L+2 15%	
9			2.87	431	0.08	H-3→L+3 62%, H-2→L+2 23%	
10	III		2.88	430	0.12	H-2→L+3 38%, H-3→L+2 36%	MLCT/ ILCT
11			3.11	399	0.04	H-5→L 70%	
12			3.15	393	0.09	H→L+4 71%, H-5→L 17%,	
13			3.27	378	1.17	H→L+5 81%	
		T1	*0.92*			*H→L 98%*	*LMCT*

Ru-HBDP	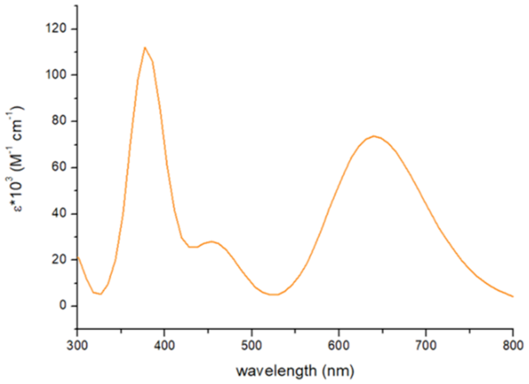		
	**Tr=1(686 nm)**	**Tr=2(591 nm)**	**Tr=3(545nm)**
**Particle**	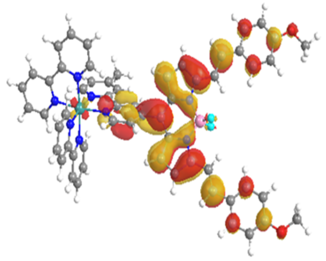	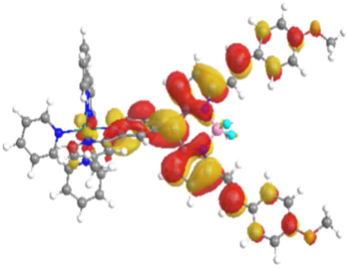	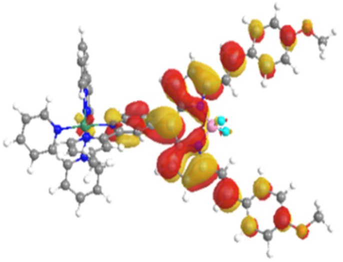
**Hole**	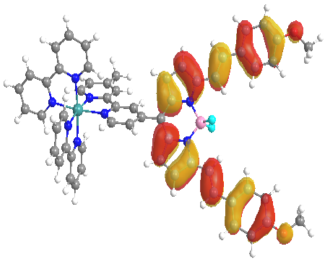	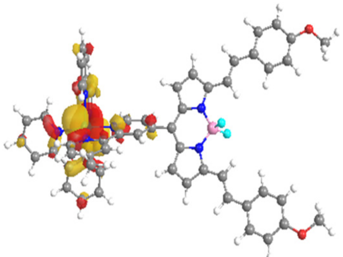	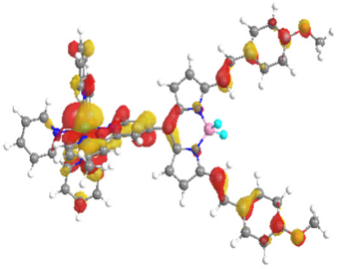
	**Tr=4(505 nm)**	**Tr=5(481 nm)**	**Tr=6(477 nm)**
**Particle**	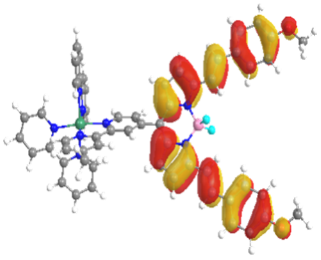	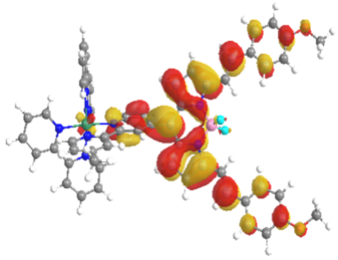	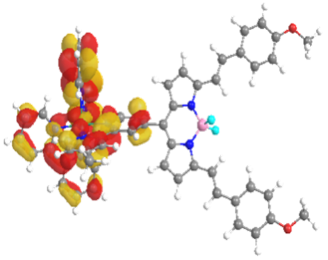
**Hole**	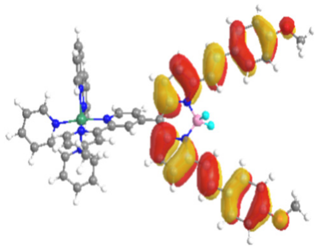	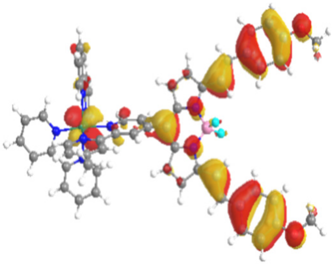	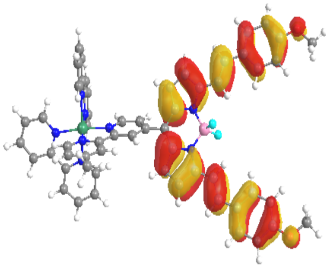

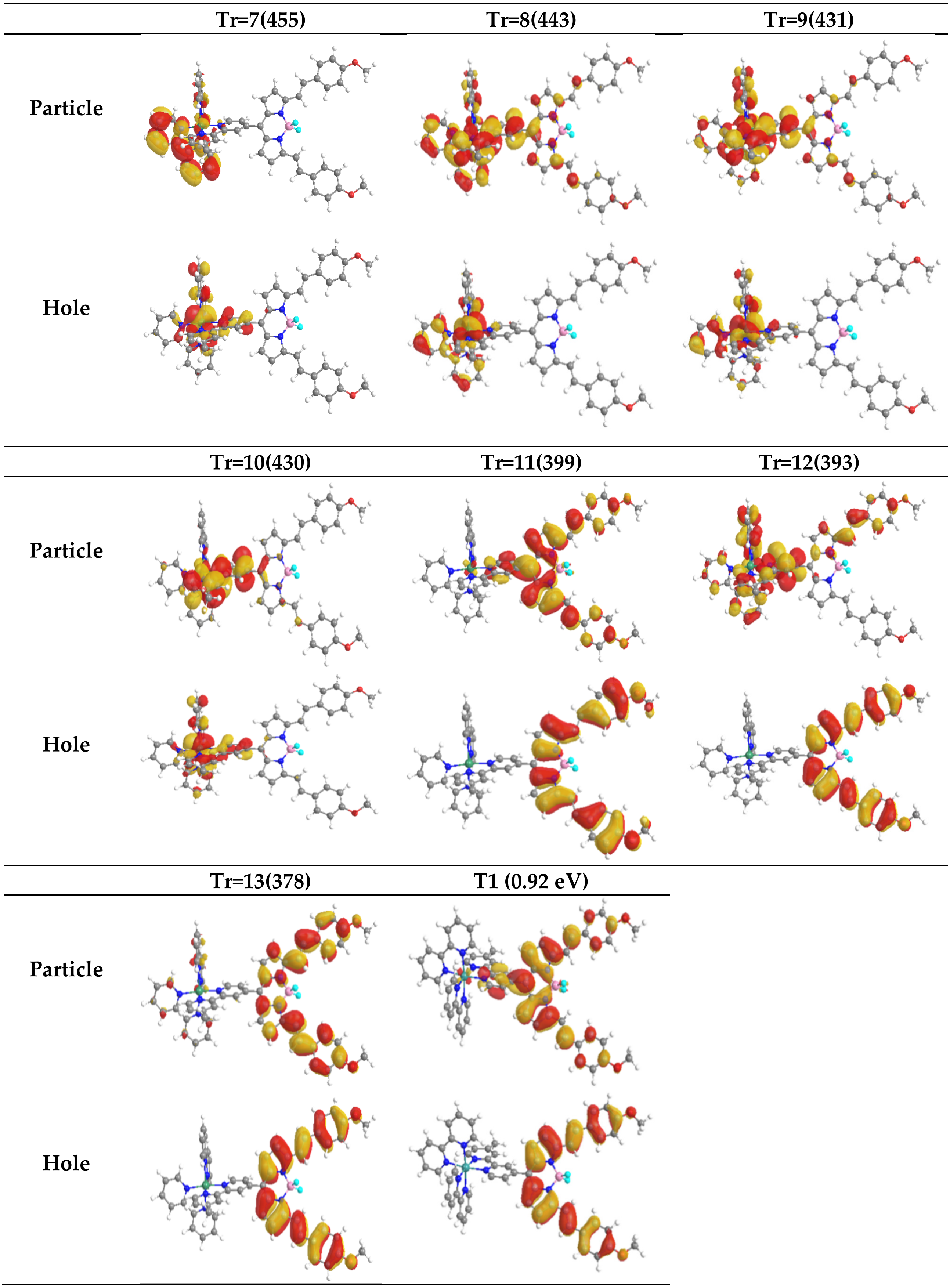
Tr=7(455)
Tr=8(443)
Tr=9(431)
Particle
Hole
Tr=10(430)
Tr=11(399)
Tr=12(393)
Particle
Hole
Tr=13(378)
T1 (0.92 eV)
Particle
Hole

Figure S6

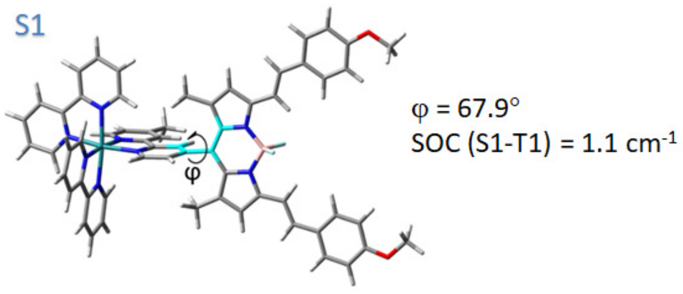

Figure S7

References

1. Qiao, L.; Liu, J.; Han, Y.; Wei, F.; Liao, X.; Zhang, C.; Xie, L.; Ji, L.; Chao, H. Rational Design of a Lysosome-Targeting and near-Infrared Absorbing Ru(II)–BODIPY Conjugate for Photodynamic Therapy. *Chem. Commun.* **2021**, *57*, 1790–1793.
